# Hospital Expenditure.—The Commissariat

**Published:** 1896-08-22

**Authors:** 


### The Institutional Workshop.

### HOSPITAL EXPENDITURE.—THE COMMISSARIAT.

### XXIV.—THE COST OF MILK.

In further illustration of the difference existing in hospitals with reference to the consumption of milk, the following tables may be studied, giving the cost per head at some leading institutions in London and elsewhere for this item :—

| | Daily Average of Patients. | Number Boarded of Staff and Household. | Average Yearly Cost of Milk per Inmate. |
|---|---|---|---|
| London Hospitals— | | | |
| St. George's | 323 | 183 | £2 3 0 |
| Great Northern Central | 68 | 38 | 2 6 0 |
| University | 186 | 54 | 2 8 0 |
| Charing Cross | 150 | 92 | 2 12 0 |
| Royal Free | 112 | 70 | 3 0 0 |
| St. Mary's | 251 | 122 | 3 0 0 |
| Middlesex | 215 | 141 | 3 1 0 |
| Seamen's | 214 | 69 | 3 3 0 |
| Guy's | 411 | 35 | 3 6 0 |
| Metropolitan | 47 | 50 | 3 6 0 |
| London | 656 | 77 | 3 9 0 |
| Westminster | 176 | 48 | 4 2 0 |
| St. Thomas's | 365 | 46 | 4 9 0 |
| Provincial Hospitals— | | | |
| Leeds General Infirmary | 355 | 159 | 1 18 0 |
| Norfolk and Norwich | 135 | 64 | 2 0 0 |
| Bristol General | 172 | 90 | 2 5 0 |
| Oxford Radcliffe | 105 | 69 | 2 6 0 |
| Hull Royal Infirmary | 160 | 63 | 2 6 0 |
| Sussex County | 134 | 75 | 2 8 0 |
| Newcastle-on-Tyne | 256 | 111 | 2 12 0 |
| Sheffield General Infirmary | 178 | 78 | 2 13 0 |
| Birmingham General | 231 | 125 | 2 14 0 |
| Birmingham Queen's | 113 | 55 | 2 14 0 |
| York County | 103 | 42 | 2 16 0 |
| Cambridge, Adenbrooke's | 56 | 56 | 3 4 0 |
| Halifax | 92 | 41 | 3 4 0 |
| Scotch & Irish Hospitals— | | | |
| Glasgow Royal Infirmary | | | 1 16 0* |
| Aberdeen | | | 2 4 0 |
| Kilmarnock | | | 2 6 0 |
| Ayr | | | 2 8 0 |
| Edinburgh Royal Infirmary | | | 2 15 0* |
| Glasgow Western Infirmary | | | 2 16 0 |
| Belfast Royal | | | 3 6 0 |

* Including butter-milk, &c.

To appreciate the full value of these figures it must be remembered that the amount of milk prescribed in the diet tables appears to be almost identical; and although, as we have seen, a high proportion of patients on low diet necessitates increased consumption of milk, it does not appear that the highest figures, by any means, are reached for this item by hospitals where this increased consumption might be expected. The importance of these apparently causeless variations in cost may be best appreciated by the consideration that every additional shilling per head represents £5 a year for every 100 inmates; so that, in the case of a large institution, the difference of £2 a head which appears in the above tables may amount to something like £700 or £800 a-year for this one item alone.

In no direction is it more difficult to check consumption, so cheap is the article, and so necessarily free the supply. Consequently, in no direction is it more easy for waste to occur. The want of a proper tiled larder reserved for such articles as butter, milk, and eggs is in many large London hospitals a serious deficiency and occasion of loss. When, as often happens, the milk has to be brought direct into the kitchen, and must stand there in open pans exposed to all the heat and dust of continual traffic and cooking until removed to the wards, it is hardly possible that it should not sometimes in close weather become so far deteriorated as to "turn off" before it can be used, and a very large proportion of the waste that does occur is due to the difficulty of keeping the milk perfectly sweet.

Waste, again, is largely due to the natural dread, on the part of those in charge of the patients' interests, of running short in such a needful commodity. Everyone knows the inevitable delay and uncertainty in any institution in sending down for more, and hence the sister-in-charge is liable to give the order for a good deal more than she requires in her ward, so as to cover the risk of requiring more than she gets. In one cleverly-managed hospital this trait is so well understood that several quarts of milk less than the order are invariably sent up to certain wards under extravagant sisters, who remain in blissful ignorance that their supply is daily cut down. Again, the little extra quarter of a cup poured out for each patient, perhaps, by the lavish hand of the probationer, and very likely left over to be thrown away, may easily be responsible for a yearly waste amounting in the aggregate to as much as £100. Careful measurement of quantities, strict reckoning of requirements, and the most scrupulous attention to the storage of the milk supply can alone prevent waste in this direction, and the savings which can be effected by such measures, without in any degree curtailing the comfort of the inmates, are so considerable as to be well worth the attention of every manager.

But the responsibility of the hospital with regard to its milk supply does not begin with the appearance of the milk in the hospital larder. It reaches farther back, even beyond the contractor, to the numerous farms whence the supply is drawn, for a recent trial has shown that the contractor can neither be trusted to ensure that the milk he buys is perfectly satisfactory, nor even, when this happens to be the case, to deliver it in the same condition of purity as when he received it. That the hospital manager should be expected personally to inspect the sources of the milk supply will probably be considered an altogether fantastic requirement. Yet it should be remembered that impure milk is becoming increasingly manifest as a source of danger in spreading the germs, not only of infectious complaints, but of such subtle maladies as tuberculosis. The danger, which is minimised in persons of ordinary health by the variety of their diet and the natural tendency to destruction of disease germs under healthy conditions, is greatly intensified when the diet consists solely or in great part of milk, and the consumer is in an enfeebled condition. The hospital, moreover, gets its milk very cheap, for very little if at all more than half the price paid by the ordinary consumer, and is therefore exposed to the increased risk of adulteration or careless storage which invariably attends the purchase of all articles in bulk at a low price.

The only effectual remedy lies, as we pointed out in the case of the meat supply, in co-operation among the various metropolitan institutions. A co-operative hospital store could alone supersede the present unsatisfactory contract system, procure a sufficient supply of milk from farms under the direct inspection of a qualified official, and furnish that reliable guarantee of the satisfactory condition of all cows and dairies concerned, which is now practically unattainable. Few persons have any idea of the variations in quality presented by the milk of different cows, baffling scientific analysis and rendering adulteration in some instances almost impossible of detection. It is clear from the different prices paid for milk by London hospitals that a varying standard as to quality exists among hospital authorities. But the public will not be satisfied that anything short of the very best should be procured in this particular, and the attainment of this requirement at a reasonable rate can be managed by co-operation alone.

The sterilisation of milk which is now fast growing in favour is commonly carried out roughly in each institution as required. This might with far greater certainty and propriety be effected in proper premises by trained hands, such as could be afforded only in a co-operative store supplying many institutions.

